# Assessing Gait Function in Lower Limb Rehabilitation: The Role of the Gait Analysis and Motion Score (GAMS)

**DOI:** 10.3390/jcm14124376

**Published:** 2025-06-19

**Authors:** Walter Bily, Ferdinand Prüfer, Klemens Adamer, Roman Lederwasch, Špela Matko, Michael J. Fischer, Vincent Grote

**Affiliations:** 1 Department of Physical Medicine and Rehabilitation, Clinic Ottakring, Vienna Healthcare Group, 1160 Vienna, Austria; 2 Ludwig Boltzmann Institute for Rehabilitation Research, 1140 Vienna, Austria; 3 Rehabilitation Centre Kitzbühel, 6370 Kitzbühel, Austria; 4 Institute for Outcomes Research, Medical University of Vienna, 1090 Vienna, Austria; 5 Department of Orthopaedics and Trauma, Medical University of Graz, 8036 Graz, Austria

**Keywords:** gait analysis, rehabilitation, lower extremity, reproducibility of results, outcome assessment, healthcare

## Abstract

**Background:** Assessment of gait function is crucial for optimising rehabilitation outcomes. The gait analysis and motion score (GAMS) summarises qualitative and quantitative gait parameters from treadmill-based analyses to evaluate functional walking status. **Objectives:** To assess the sensitivity of the GAMS for detecting short-term changes, its test–retest reliability, and its correlation with the Western Ontario and McMaster Universities Osteoarthritis Index (WOMAC) and the Timed Up and Go (TUG) test. **Methods:** A retrospective analysis of 94 inpatient rehabilitation patients with hip, knee, or ankle impairments was performed. Changes in GAMS, WOMAC, and TUG scores and their interrelationships were assessed at both admission and discharge. **Results:** GAMS, WOMAC, and TUG showed significant improvements over time, with medium effect sizes (η^2^ = 0.303 to 0.434; *p* < 0.001). No significant differences in outcome measures were observed between groups. Moderate to strong correlations were found between pre- and post-rehabilitation scores for GAMS, TUG, and WOMAC (*r* = 0.58 to *r* = 0.90), indicating good test–retest reliability. A significant low negative correlation between GAMS and TUG was observed for all patients at admission (*r* = −0.30, *p* = 0.003) and discharge (*r* = −0.26, *p* = 0.030). No significant correlations were observed between GAMS and WOMAC in any patient group. Baseline GAMS scores significantly influenced change scores. **Conclusions:** GAMS is a sensitive and reliable tool for detecting short-term changes in gait parameters. GAMS and TUG assess related but distinct constructs, with GAMS and WOMAC assessing different domains of gait function. Therefore, GAMS provides complementary information not captured by WOMAC or TUG.

## 1. Introduction

Correcting gait abnormalities and improving walking function are primary objectives in the rehabilitation of patients with impaired mobility. Accurate assessment and continuous monitoring of gait are critical for identifying abnormalities that may hinder mobility and recovery. A precise analysis of gait dysfunction is essential for designing targeted interventions to improve rehabilitation outcomes.

In orthopaedic rehabilitation, patient-reported outcome measures (PROMs) and performance-based measures are often used to assess improvements in function and physical performance. PROMs are standardised assessments that capture a patient’s self-reported health, symptom or functional status, such as the Western Ontario and McMaster Universities Osteoarthritis Index (WOMAC). Performance-based measures assess a patient’s capacity in performing specific tasks that are relevant to their daily life and reflect their functional abilities (e.g., mobility, walking), such as the Timed Up and Go (TUG) test. However, both PROMs and performance-based measures can be influenced by pain perception, psychosocial factors, or compensatory strategies, which limits their ability to accurately quantify gait quality and walking biomechanics. In contrast, clinician-reported outcome measures (CROMs) rely on a healthcare professional’s clinical judgment and observation (e.g., observational gait scores), providing an expert perspective on a patient’s health or functional status.

Visual gait analysis (VGA) provides a subjective assessment based on video recordings and clinical scoring systems. While computer-based image analysis can improve the process, VGA is less sensitive, specific, valid, and reliable than instrumented gait analysis (IGA) [1]. IGA systems are essential tools for obtaining consistent, accurate data on gait abnormalities in clinical settings.

IGA using three-dimensional (3D) motion capture systems, whether overground or on a treadmill, shows high accuracy, concurrent validity, and reliability in assessing spatiotemporal and kinematic parameters, especially for hip and knee movements in the sagittal plane [2,3]. However, 3D motion analysis requires significant time and resources, limiting its use to specific clinical scenarios such as neurological conditions (e.g., cerebral palsy, Parkinson’s disease, stroke), osteoarthritis, post-surgical assessments, and haemophilia management [3]. As a result, the routine clinical use of 3D gait analysis remains impractical in many rehabilitation settings.

Although 3D gait analysis is the gold standard for gaining detailed gait insights, the complexity of the data requires summary metrics to represent qualitative and quantitative aspects of gait. Such metrics are applied either to specific gait variables or to combine multiple aspects into a composite performance score [4,5].

Existing VGA tools are designed mainly to examine neurological conditions, but do not fully address orthopaedic rehabilitation needs. Thus, a reliable gait assessment tool is needed to detect gait abnormalities and track short-term rehabilitation-related changes.

The gait analysis and motion score (GAMS) was developed to meet orthopaedic rehabilitation needs by integrating both qualitative and quantitative gait parameters [6]. As a multidimensional assessment tool, GAMS combines technical sensor data (from treadmills and pressure platforms) with clinician-rated observational video parameters, offering a comprehensive overview of gait without requiring advanced or costly motion-capture laboratories. Following a brief instruction phase, it enables a relatively easy-to-apply method of gait analysis, focusing on specific gait parameters rather than general function performance alone. GAMS enhances gait evaluation in patients undergoing rehabilitation for conditions such as hip, knee, and ankle impairments. It has demonstrated internal consistency and reliability, but its ability to supplement PROMs and CROMs is unclear.

Our objectives were to assess the sensitivity of GAMS in detecting short-term changes in gait, its test–retest reliability, and its relationship with the WOMAC and the TUG test. Additionally, this study investigated the potential of GAMS to offer supplementary clinical information beyond traditional outcome measures.

## 2. Materials and Methods

### 2.1. Participants

In this retrospective cross-sectional study, the medical records of 94 rehabilitation patients treated at the Rehabilitation Centre Kitzbühel (Kitzbühel, Austria) were analysed between January 2018 and April 2021. Patients with lower limb impairments and complete datasets for the GAMS, WOMAC, and TUG were included. The 94 patients were categorised into three groups based on their diagnosis and functional impairments: hip, knee, or ankle conditions. The hip group comprised 68% total hip arthroplasty patients; other diagnoses included pelvic fractures, gluteal muscle tears, labral lesions, and femoral neck or proximal femur fractures (ICD-10 M16, T91, T93). The knee group contained 29% who had undergone arthroscopy; the remainder were treated for knee arthroplasty, lower-leg muscle tears, patella or proximal tibial fractures, and anterior cruciate ligament (ACL) ruptures (ICD-10 M17, M23, T93). The ankle group comprised 59% ankle fracture cases; other conditions included Achilles tendinitis, ankle arthroscopy, calcaneal spurs, distal fibula or tibia fractures, osteochondritis dissecans, ankle sprains, and post-traumatic ankle osteoarthritis (ICD-10 M19, M21.4, M93.27, S82.4, T93, T94.1). In 87% of cases rehabilitation targeted unilateral, single-joint pathology; a small subset had bilateral involvement (four with bilateral ankle fractures, four with bilateral hip osteoarthritis, three with bilateral knee osteoarthritis, and one with bilateral ACL ruptures). All patients underwent comprehensive outcome assessments at admission and after three weeks of inpatient rehabilitation. Demographic data are shown in Table 1.

All patients participated in a three-week, individualised rehabilitation programme totalling at least 1800 min of therapy, with 2–3 h of daily therapy. Treatment followed Austrian guidelines for rehabilitation programmes [7], ensuring each patient’s activities were tailored to their specific health and functional status. The programme included individual physiotherapy, medical exercise therapy, aquatic exercise, endurance training (cycling and Nordic walking), and strength and balance exercises. Additionally, it included health education, lifestyle coaching, medical massage, electrotherapy, ultrasound therapy, and stretching/relaxation.

The study was conducted in accordance with the principles outlined in the Declaration of Helsinki and was approved by the Ethics Committee of the Medical University of Innsbruck (MUI EK.nr.: 1196/2021). The study was registered at the German Clinical Trials Register (DRKS-ID DRKS00026113). Study reporting followed the STROBE guidelines for observational studies.

### 2.2. Testing and Rating Procedure

Video recorded gait analysis was conducted using a Zebris FDM-T treadmill system (h/p/cosmos mercury med) with two SYNCLightCam modules and an integrated pressure plate (Zebris Medical GmbH, Isny, Germany). The treadmill had a running surface of 210 × 80 × 137 cm and a belt speed of 0–22 km/h, adjustable in 0.1 km/h increments. The pressure plate contained 3432 sensors over a 111.8 × 49.5 cm area, with a sampling rate of 120 Hz and a sensor resolution of 0.6 sensors/cm^2^. The sensor threshold was set at 1 N/cm^2^. The Zebris system is commonly used in orthopaedic, neurologic, and geriatric rehabilitation to analyse movement disorders and assess kinematic gait parameters at different walking speeds and across multiple gait cycles. Temporospatial gait parameter reliability measured with the Zebris system in healthy seniors showed high ICC values of 0.86–0.97 [8].

Participants wore tight shorts to allow joint angle assessments and walked barefoot on a horizontal treadmill (0% incline). A familiarisation period of 1–3 min was provided based on individual’s experience and skill. The treadmill speed started at 0.5 km/h, gradually increasing in 0.5 km/h increments until participants reached a comfortable walking pace. Gait data were recorded for 30 s after 3 min of walking, capturing an average of 52 ± 2 steps. A second trial was allowed if the first attempt was unsuccessful. An experienced orthopaedic technician analysed kinematic data from video sequences, while the Zebris treadmill report yielded temporospatial data. The orthopaedic technician was blinded to group allocation to reduce observer bias.

GAMS is a clinician-reported outcome measure designed for gait assessment in orthopaedic patients. It consists of 10 observational and 5 measured items, scored as either 0 (abnormal) or 1 (normal). The total GAMS score ranges from 0 to 25, with lower scores indicating more severe gait abnormalities. Ten parameters are rated bilaterally, contributing to the overall score. GAMS has shown excellent inter-rater reliability (ICC 0.97–0.98) and a rating agreement of 82.5–86.1% [6].

The WOMAC is a validated patient-reported outcome measure originally developed for knee osteoarthritis but widely used in orthopaedic rehabilitation. It includes 24 items that assess pain, stiffness, and functional deficits in the lower limb, with scores ranging from 0 (best) to 10 (worst). The sum score was used for statistical analysis. WOMAC has proved to be a reliable and responsive tool for evaluating patient perspectives in rehabilitation [9].

The TUG test developed by Podsiadlo in 1991 [10] is a widely used clinical tool for assessing general mobility and fall risk. Participants wearing sports shoes sat in a standard armchair (seat height: 46 cm) with armrests. They were instructed to rise, walk three meters, turn around, walk back, and sit down as quickly as possible. The time from losing contact with the backrest to sitting down was recorded with a stopwatch (precision: ±0.1 s). Armrest use was allowed, and no participant needed walking aides. The TUG test has shown excellent reliability in similar patient populations (ICC 0.95–0.97) [11].

### 2.3. Statistical Analysis

Data analysis was conducted using IBM SPSS Statistics for Windows (v.29; IBM, Chicago, IL, USA). Patients were classified into three main groups: (1) hip conditions, (2) knee conditions, and (3) ankle conditions. We calculated score differences (Δ) between the beginning (t1) and the end (t2) of rehabilitation. Effect sizes (Cohen’s *d*) for within-subject designs were determined and interpreted based on those of Sawilowsky [12], with the following classifications: very small (0.01), small (0.2), medium (0.5), large (0.8), very large (1.2), and huge (2.0).

To compare differences within and between groups, we employed a 3 × 2 ANOVA, with group (hip, knee, ankle) as between-subject and time (pre- and post-rehabilitation) as within-subject factors. Effect sizes were reported as partial eta squared (η*_p_*^2^) and interpreted following Cohen [13]: 0.01 (small), 0.06 (medium), and 0.14 (large). Post-hoc analysis was performed using multiple *t*-tests (Fisher’s LSD test).

We assessed the test–retest reliability of the GAMS by calculating the pre–post correlation from admission to discharge using Pearson’s correlation. Additionally, we analysed the relationships between GAMS, WOMAC, and TUG scores, performing correlation analyses across both time points, and checked for changes in values. The relationship strength for Pearson’s correlation coefficient was interpreted according to Hinkle et al. [14] (*r* < 0.5 low, *r* = 0.5–0.7 moderate, and *r* > 0.7 high correlation).

To adjust for differences in baseline values among groups, we applied the formula T2D = t2 + (t2 − t1) to reflect individual performance, functional status, and changes relative to baseline [15,16,17]. This approach enables comparison of groups with varying baseline distributions in a pre–post design, mitigating issues like mathematical coupling or regression effects that can arise with ANCOVAs [18]. Participants were stratified into tertiles based on T2D (above average, average, below average). Finally, scatter plots with linear regression lines were created to illustrate relationships between baseline values and changes (Δ: t2 − t1) of GAMS relative to T2D scores.

## 3. Results

### 3.1. Change over Time and Differences Between Groups

Across all three outcome measures—GAMS, WOMAC, and TUG—the ANOVA demonstrated significant improvements, with large effect sizes (η^2^ of 0.303–0.434; *p* < 0.001). This is reflected by the descriptive statistics, as well as within-subject *t*-tests and corresponding effect sizes (Table 2). Patients in the hip and knee groups exhibited the most pronounced improvements, with Cohen’s *dz* values exceeding 0.6 across all outcome measures. The ankle group showed significant improvements, but its effect sizes were generally lower, particularly for WOMAC. The TUG results consistently demonstrated the highest effect sizes across groups, with the hip group reaching *dz* = 1.02 (*p* < 0.001), indicating a robust enhancement in functional mobility.

No statistically significant main effects for group or time × group interactions were identified in the ANOVA analysis for any of the outcome measures.

**Figure 1:**
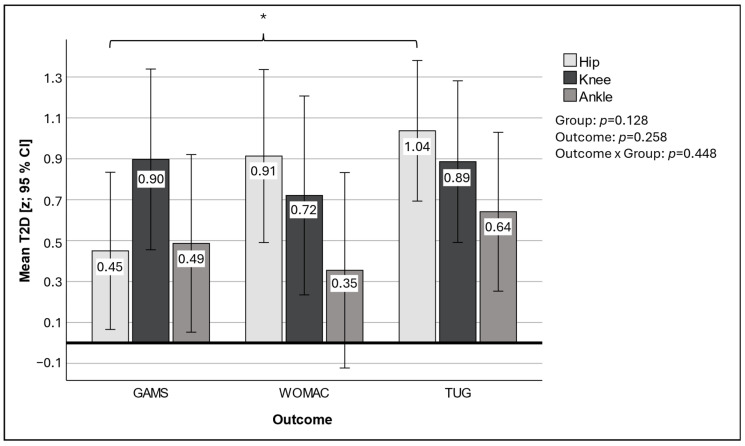
Comparison of performance using *z*-transformed T2D scores. TUG—Timed Up and Go test; WOMAC—Western Ontario and McMaster Universities Osteoarthritis Index; GAMS—gait analysis and motion score; z—*z*-transformed T2D scores, WOMAC and TUG T2D were inverted to align their scales with GAMS, ensuring that all measurements are presented in the same direction; 95% CI—error bars are at 95% confidence interval; *p*—significance level of ANOVA with group as independent variable; *—*p*-value of post hoc analysis, *p* < 0.05.

After adjusting for baseline differences, *z*-transformed T2D scores did not reveal significant differences between groups (*p* = 0.128) or outcome measures (*p* = 0.258), as illustrated in Figure 1. Nevertheless, a notable finding was that patients in the hip group demonstrated significantly better performance in the TUG than in the GAMS, with T2D scores of 1.04 and 0.45, respectively (*p* < 0.05).

### 3.2. Reliability and Relationships

Pre–post correlations of baseline and discharge values demonstrated moderate to strong and significant reliability across all groups and outcome measures (*p* < 0.001). Pre–post correlations ranged from *r* = 0.58 (WOMAC) to *r* = 0.90 (GAMS), indicating excellent test–retest reliability (Table 3).

A significant low negative correlation between GAMS and TUG was observed across all patients for admission (*r* = −0.30, *p* = 0.003) or discharge (*r* = −0.26, *p* = 0.030) scores. When analysed by group, only the hip cohort exhibited significant correlations between TUG and GAMS at admission (*r* = −0.51, *p* = 0.001) and discharge (*r* = −0.49, *p* = 0.002). Only the ankle group displayed a significant correlation between TUG and GAMS change scores (*r* = 0.43, *p* = 0.019). In contrast, no significant correlations were found between GAMS and WOMAC admission, discharge, or change scores in any group (Table A1, in Appendix A).

**Figure 2:**
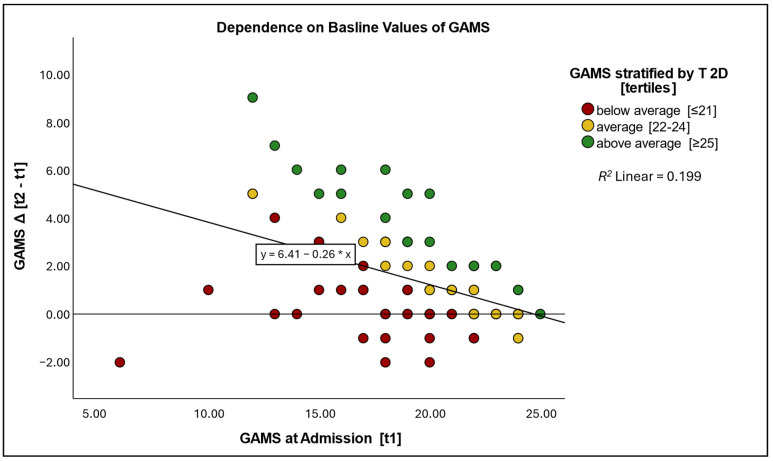
Dependence of changes on GAMS baseline value. GAMS—gait analysis and motion score; Δ [t1 − t2]—change score = discharge score—admission score; t1—time point of admission measurement; *R*^2^ Linear—*R*-squared effect size of linear regression; Pearson correlation coefficient of admission and change score for GAMS, *r* = −0.45, *p* < 0.001; for TUG, *r* = −0.63, *p* < 0.001; for WOMAC, *r* = −0.65, *p* < 0.001.

Baseline GAMS values accounted for approximately 19.9% of the variance in change scores (*R*^2^ = 0.199, *p* < 0.001) (Figure 2). The regression analysis revealed an association between higher initial impairment (lower baseline scores) and greater improvements, a pattern also observed for TUG (*r* = −0.63, *p* < 0.001) and WOMAC (*r* = −0.65, *p* < 0.001).

### 3.3. Changes in GAMS at Item Level

**Figure 3:**
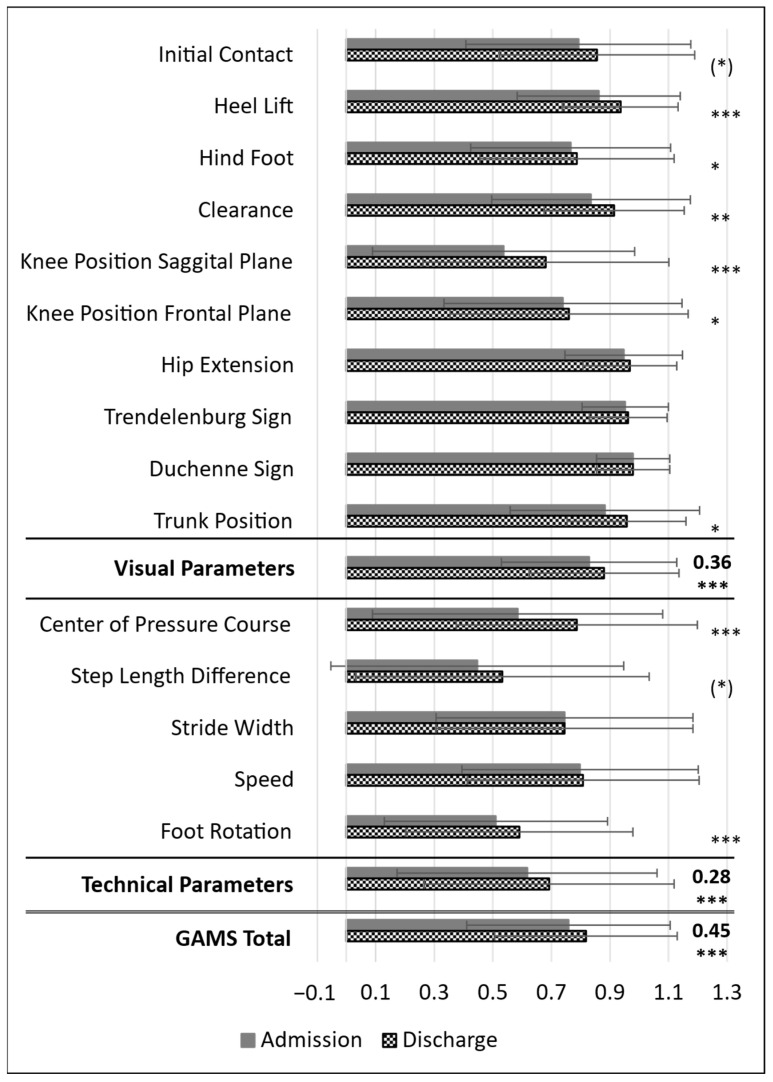
Mean GAMS values for admission and discharge on item level. GAMS—gait analysis and motion score; mean scores for admission and discharge across the 15 items, two sub-domains and total; error bars at standard deviation; first ten items classified as visual parameters, last five items classified as technical parameters; scale from 0 (indicating impairment) to 1 (indicating no impairment); effect size η*_p_*^2^ (partial eta squared) for subdomains and total score; (*)—significance of ANOVA, *p* < 0.1; *—significance of ANOVA, *p* < 0.05; **—significance of ANOVA, *p* < 0.01; ***—significance of ANOVA, *p* < 0.001.

No significant differences between groups were found for any GAMS items except the Trendelenburg sign (η*_p_*^2^ = 0.08, *p* = 0.024): at admission, 7 hip and 2 ankle patients presented with Trendelenburg, 6 hip and 1 ankle patient at discharge, and no knee patients exhibited Trendelenburg at either time. While the overall GAMS score indicated significant improvement (η*_p_*^2^ = 0.45, *p* < 0.001), an analysis at the item level revealed heterogeneity in the extent of changes (Figure 3). Some gait parameters, including centre of pressure course, knee position in the sagittal plane, heel lift, and foot rotation, improved significantly over the short rehabilitation period (*p* < 0.001). Other parameters, such as the Trendelenburg sign, Duchenne sign, stride width, and speed, remained stable or exhibited only minimal changes.

When categorised into subdomains, visual and technical gait parameters demonstrated significant improvements (η*_p_*^2^ = 0.36 and η*_p_*^2^ = 0.28, respectively; *p* < 0.001). This suggests that certain gait adaptations respond more to short-term rehabilitation, whereas others may require extended interventions for measurable improvements.

## 4. Discussion

The study aim was to assess the significance of GAMS in scoring gait function by detecting short-term changes in gait parameters, its test–retest reliability, and its relationship with WOMAC and TUG. This study also explored the potential of GAMS to provide supplementary clinical information beyond traditional outcome measures.

Our results demonstrate that the GAMS reflects rehabilitation-induced changes in gait function over a three-week inpatient stay, as we saw significant improvements in all three groups with large effect sizes (Table 2). The significant improvements in GAMS, WOMAC, and TUG observed in our sample are consistent with previous studies which showing significant improvements in functional mobility and patient-reported outcomes after orthopaedic rehabilitation [15].

Regarding the clinical relevance of the observed significant improvements in GAMS, TUG, and WOMAC, our findings suggest that changes were not only statistically significant but also meaningful in a rehabilitation context. For the TUG test, Wright et al. [19] report a minimal clinically important difference (MCID) between 0.8 and 1.4 s for orthopaedic populations. In our cohort, the hip and knee groups showed average improvements of 1.2 s, right at the midpoint of this range, indicating clinically relevant improvement. The ankle group improved by 0.8 s, meeting the lower bound of the MCID range, and thus may also be considered clinically meaningful, albeit with less certainty. For the WOMAC, Angst et al. [20] report an MCID of 17–22% improvement relative to baseline. In our sample, mean improvements were 39% in the hip group, 36% in the knee group, and 22% in the ankle group, indicating that all groups achieved clinically meaningful changes in WOMAC. For GAMS, no established MCID exists to date. Based on a distribution-based estimate (0.5 × baseline standard deviation, SD = 3.65), an approximate MCID would be 1.825 points. While the observed mean improvements in the hip (1.5 points), knee (1.4 points), and ankle (1.2 points) groups fell short of this estimate, they approached the threshold, suggesting potential clinical relevance, particularly given the short three-week rehabilitation period. Taken together, these results indicate that observed improvements, especially in TUG and WOMAC, were not only statistically robust but also likely meaningful for patients and clinicians.

We found excellent test–retest reliability for GAMS as illustrated by the significant pre–post correlations (Table 3). We also found high pre–post correlations for WOMAC and TUG in our cohort (*r* = 0.79 and *r* = 0.64, respectively; *p* < 0.001), reflecting the established reliability of WOMAC and TUG seen in multiple populations [21,22]. This confirms that GAMS can be a stable and reproducible tool for assessing gait function over short periods. The strong reliability supports its use in clinical settings, where consistent and repeatable measures are crucial for tracking patient progress.

GAMS showed a significant low negative correlation with TUG, indicating that both assess functional mobility but capture different aspects of gait function. This aligns with previous research demonstrating strong associations between TUG and spatiotemporal gait parameters such as step length, stride velocity, and stance phase duration [23] in elderly patients. Comparable to our knee group, no significant associations were observed between kinematic Gait Deviation Index (GDI) scores and TUG in knee osteoarthritis patients [4]. In our study, functional mobility improvements (TUG) were not necessarily accompanied by gait biomechanics improvements (GAMS), most prominently in the ankle group. Patients might improve their TUG scores by compensating for inefficient gait biomechanics rather than by correcting their underlying gait patterns. However, other reasons such as exercise induced improvement in walking fitness or reduced pain perception cannot be ruled out. This suggests that GAMS provides additional insights into gait biomechanics beyond what TUG captures, making it a valuable complementary tool in rehabilitation assessment and management.

No significant correlation was found between GAMS and WOMAC. This result is consistent with previous studies, which report weak to moderate associations between patient-reported outcome measures (PROMs) such as WOMAC, Knee Society score (KSS), Hospital for Special Surgery (HSS) score, and gait analysis parameters, particularly in patients with knee osteoarthritis or after total knee arthroplasty [24,25,26,27,28,29,30,31,32].

The lack of correlation suggests that PROMs and CROMs can be used to assess different functional aspects. WOMAC measures pain, stiffness, and self-perceived physical function, whereas GAMS evaluates objective gait biomechanics. Patients may report lower WOMAC scores (indicating less pain and better function) but still exhibit gait abnormalities, possibly due to compensation mechanisms. Conversely, some patients may show persistent pain or stiffness despite improvements in gait biomechanics. These findings highlight the importance of using both PROMs and CROMs to gain a more holistic view of patient function during rehabilitation.

The individual GAMS items displayed varied responsiveness over the rehabilitation period: centre of pressure, sagittal knee position, heel lift, and foot rotation improved significantly (Figure 3), while Duchenne sign and stride width remained unchanged and speed and Trendelenburg sign showed minimal change. High baseline scores for hip extension, Trendelenburg sign, and Duchenne sign (0.95, 0.95, 0.98) indicate ceiling effects, suggesting these items may be more useful for early impairment detection or targeting interventions rather than for monitoring progress. The larger gains in visual parameters align with clinical observations that observable gait abnormalities often resolve before spatiotemporal metrics shift substantially.

These findings imply that rehabilitation should target those modifiable gait parameters that demonstrated responsiveness and are not prone to ceiling effects, such as initial contact pattern, heel lift, ankle position, clearance, knee position, trunk position, step length symmetry, and weight acceptance or loading response reaction (e.g., centre of pressure course). Interventions might include stride-length drills to improve step symmetry, targeted dorsiflexor strengthening to enhance initial contact, and visual or verbal feedback to normalize foot placement and trunk posture. In contrast, static parameters (hip extension, Trendelenburg sign, Duchenne sign) may help flag patients with more complex impairments requiring additional support, rather than serving as indicators of short-term progress. Recognising which gait components might improve can help clinicians optimise therapy and allocate resources effectively. The item-level GAMS provides valuable insights into which gait parameters change with rehabilitation, allowing for more targeted approaches.

The significant negative correlation between the initial and change GAMS (*r* = −0.45, *p* < 0.001), TUG (*r* = −0.63, *p* < 0.001), and WOMAC (*r* = −0.65, *p* < 0.001) suggests that change scores depend on baseline values. This indicates patients with more severe gait impairments at admission experienced greater improvements over the three-week rehabilitation period (Figure 3).

These results highlight the importance of considering baseline function when interpreting rehabilitation outcomes: patients with lower initial scores often improve more, but those with higher baseline function can still benefit despite modest change scores. Setting individualised rehabilitation goals can be beneficial, where success is measured by both absolute score changes and clinically meaningful functional improvements.

Owing to its retrospective, single-centre design, causal inferences between changes in GAMS, TUG, and WOMAC are limited and generalizability of our findings to other patient populations or outpatient settings may be constrained. The exclusion of records with incomplete datasets may have introduced selection bias. Although the orthopaedic technician was blinded to group allocation, the measurements being conducted over the natural course of rehabilitation meant that blinding of timepoint was not feasible, possibly introducing rater bias, which might have influenced the scoring of the video analysis. Moreover, the study population consisted exclusively of patients with orthopaedic conditions affecting the lower extremities (hip, knee, and ankle), which limits the applicability of our findings to other clinical populations.

Future studies should validate GAMS in larger, diverse cohorts and assess its utility for long-term monitoring. Further research is needed to evaluate whether GAMS is suitable for use in patients with neurological gait disorders, such as Parkinson’s disease or stroke.

## 5. Conclusions

Certainly, each outcome measure reflects a distinct gait function and performance domain. Improvements in one specific domain do not necessarily correlate with changes in other domains. GAMS and TUG, as CROMs, assess related but distinct functional mobility and gait constructs, while WOMAC, as a PROM, appears to capture different functional aspects. Therefore, the use of a combination of these measures helps to assess changes across specific domains. The GAMS enables individualised assessment and monitoring of functional mobility and gait biomechanics during rehabilitation, serving both as a measure of change and a valuable screening tool for identifying patients with specific gait deficits. Rehabilitation professionals can combine item-level analysis with baseline-adjusted change scores, thus tailoring interventions to facilitate personalised, targeted rehabilitation.

## Figures and Tables

**Table 1:** Demographic data of subjects in all three groups.

	Hip	Knee	Ankle
*n* (m/f)	37 (19/18)	28 (12/16)	29 (13/16)
Age	55.9 ± 10.4	52.1 ± 14.7	48.0 ± 11.7
Height	173.7 ± 11.3	171.7 ± 8.0	172.9 ± 9.1
Weight	82.7 ± 20.2	83.1 ± 18.1	80.3 ± 18.1
BMI	27.4 ± 5.5	28.0 ± 4.7	26.8 ± 5.3

*n*—number of patients; m/f—male/female; age, height, weight and BMI—Mean ± SD.

**Table 2:** Outcome measures at baseline (t1), changes (t2 − t1, Δ) and effect sizes (Cohen’s *dz*).

	Hip			Knee			Ankle		
	t1	Δ	C*dz*	t1	Δ	C*dz*	t1	Δ	C*dz*
GAMS	18.6 ± 3.7	1.5 ± 2.4	0.63 ***	20.1 ± 3.6	1.5 ± 2.1	0.73 ***	19.4 ± 3.7	1.2 ± 1.8	0.64 **
WOMAC	39.6 ± 31.7	−15.5 ± 25.9	0.60 ***	54.9 ± 39.1	−19.9 ± 28.4	0.70 ***	49.2 ± 39.9	−10.9 ± 32.8	0.33 (*)
TUG	8.0 ± 2.0	−1.2 ± 1.1	1.02 ***	8.4 ± 2.0	−1.2 ± 1.6	0.77 ***	8.1 ± 2.1	−0.8 ± 1.0	0.88 ***

Mean ± standard deviation; t1—time point of admission measurement; Δ—change from admission to discharge (Δ = discharge score − admission score); C*dz*—effect size Cohen’s *d*; TUG—Timed Up and Go test; WOMAC—Western Ontario and McMaster Universities Osteoarthritis Index; GAMS—gait analysis and motion score; (*)—*p*-value of dependent *t*-test *p* < 0.1, **—*p*-value of dependent *t*-test *p* < 0.01, ***—*p*-value of dependent *t*-test *p* < 0.001.

**Table 3:** Pre–post correlations from admission to discharge.

Pre–Post	GAMS	WOMAC	TUG
Hip	0.76 ***	0.58 ***	0.82 ***
Knee	0.82 ***	0.70 ***	0.60 ***
Ankle	0.90 ***	0.61 ***	0.89 ***
ALL	0.82 ***	0.64 ***	0.79 ***

TUG—Timed Up and Go test; WOMAC—Western Ontario and McMaster Universities Osteoarthritis Index; GAMS—gait analysis and motion score; Pre–Post—test–retest reliability from admission to discharge ***—significance of correlation, *p* < 0.001.

## Data Availability

Research data supporting this publication are stored at our institutional digital data repository for published research, accessible at https://creed.lbg.ac.at/ (accessed on 2 May 2025). The datasets analysed in this manuscript are not publicly available due to ethical and legal restrictions, as they contain potentially identifying and sensitive patient information, but pseudonymised datasets have been created for the purpose of reuse, accessible at creed.lbg.ac.at. Please direct requests for access to anonymised datasets to the corresponding author.

## Abbreviations

The following abbreviations are used in this manuscript:

ACL	Anterior cruciate ligament
CROM	Clinician-reported outcome measure
GAMS	Gait analysis and motion score
GDI	Gait deviation index
HSS	Hospital for Special Surgery score
IGA	Instrumented gait analysis
KSS	Knee Society score
MCID	Minimal clinically important difference
PROM	Patient-reported outcome measure
TUG	Timed Up and Go test
VGA	Visual gait analysis
WOMAC	Western Ontario and McMaster Universities Osteoarthritis Index
3D	Three-dimensional

## Appendix A

**Table A1:** Relationships of GAMS with WOMAC and TUG by group.

		Admission		Discharge		Changes	
		TUG	WOMAC	TUG	WOMAC	TUG	WOMAC
GAMS	Hip	−0.512 ***	0.124	−0.491 **	0.072	−0.267	0.006
	Knee	−0.212	−0.236	−0.191	−0.099	−0.281	−0.224
	Ankle	−0.180	0.114	−0.102	0.124	0.433 *	0.062
	ALL	−0.299 **	0.038	−0.225 *	0.073	−0.132	−0.051

Pearson correlations coefficient for GAMS with TUG and WOMAC at admission, discharge and for change values; TUG—Timed Up and Go test; WOMAC—Western Ontario and McMaster Universities Osteoarthritis Index; GAMS—gait analysis and motion score; *—significance of correlation, *p* < 0.1; **—significance of correlation, *p* < 0.05; ***—significance of correlation, *p* < 0.001.

**Table A2:** GAMS at Item level.

Item	Admission	Discharge	Δ	*p*	η_p_^2^	T2D	Side Difference (*p*-Value)	Group Difference
Initial Contact	0.79 ± 0.38	0.86 ± 0.33	0.06 ± 0.35	0.051	0.04 (*)	0.92	0.596 (η_p_^2^ = 0.00)	0.385 (η_p_^2^ = 0.02)
Heel Lift	0.86 ± 0.28	0.94 ± 0.20	0.07 ± 0.26	0.000	0.13 ***	1.01	0.184 (η_p_^2^ = 0.02)	0.181 (η_p_^2^ = 0.04)
Hind Foot	0.77 ± 0.34	0.79 ± 0.33	0.02 ± 0.14	0.045	0.04 *	0.81	0.289 (η_p_^2^ = 0.01)	0.172 (η_p_^2^ = 0.04)
Clearance	0.84 ± 0.34	0.91 ± 0.24	0.08 ± 0.27	0.002	0.10 **	0.99	0.356 (η_p_^2^ = 0.01)	0.239 (η_p_^2^ = 0.03)
Knee Position Sagittal Plane	0.54 ± 0.45	0.68 ± 0.42	0.14 ± 0.40	0.000	0.15 ***	0.82	0.693 (η_p_^2^ = 0.00)	0.764 (η_p_^2^ = 0.01)
Knee Position Frontal Plane	0.74 ± 0.41	0.76 ± 0.41	0.02 ± 0.15	0.045	0.04 *	0.78	0.726 (η_p_^2^ = 0.00)	0.857 (η_p_^2^ = 0.00)
Hip Extension	0.95 ± 0.20	0.97 ± 0.16	0.02 ± 0.18	0.208	0.02	0.99	0.057 (η_p_^2^ = 0.04)	0.674 (η_p_^2^ = 0.01)
Trendelenburg Sign	0.95 ± 0.15	0.96 ± 0.13	0.01 ± 0.07	0.158	0.02	0.97	0.468 (η_p_^2^ = 0.01)	0.024 (η_p_^2^ = 0.08)
Duchenne Sign	0.98 ± 0.13	0.98 ± 0.13	0.00 ± 0.00	/	/	0.98	1.000 (η_p_^2^ = 0.00)	0.551 (η_p_^2^ = 0.01)
Trunk Position	0.88 ± 0.32	0.96 ± 0.20	0.07 ± 0.30	0.019	0.06 *	1.03	/	0.943 (η_p_^2^ = 0.00)
**Visual Parameters**	0.83 ± 0.30	0.88 ± 0.25	0.05 ± 0.21	0.000	0.36 ***	0.93	/	0.363 (η_p_^2^ = 0.12)
Centre of Pressure Course	0.59 ± 0.50	0.79 ± 0.41	0.20 ± 0.40	0.000	0.20 ***	0.99	/	0.130 (η_p_^2^ = 0.04)
Step Length Difference	0.45 ± 0.50	0.53 ± 0.50	0.09 ± 0.48	0.088	0.03 (*)	0.62	/	0.469 (η_p_^2^ = 0.02)
Stride Width	0.74 ± 0.44	0.74 ± 0.44	0.00 ± 0.33	1.000	0.00	0.74	/	0.216 (η_p_^2^ = 0.03)
Speed	0.80 ± 0.40	0.81 ± 0.40	0.01 ± 0.18	0.566	0.00	0.82	/	0.630 (η_p_^2^ = 0.01)
Foot Rotation	0.51 ± 0.38	0.59 ± 0.39	0.08 ± 0.34	0.001	0.10 ***	0.67	0.041 (η_p_^2^ = 0.04)	0.715 (η_p_^2^ = 0.01)
**Technical Parameters**	0.62 ± 0.44	0.69 ± 0.43	0.08 ± 0.35	0.000	0.28 ***	0.77	/	0.347 (η_p_^2^ = 0.06)
**ALL**	0.76 ± 0.35	0.82 ± 0.31	0.06 ± 0.26	0.000	0.45 ***	0.88	0.260 (η_p_^2^ = 0.13)	0.454 (η_p_^2^ = 0.16)

Results of ANOVA at GAMS item level; Δ—absolute delta (discharge—admission value); *p*—significance of ANOVA; η_p_^2^—effect size partial eta squared of ANOVA; (*)—significance of ANOVA, *p* < 0.1; *—significance of ANOVA, *p* < 0.05; **—significance of ANOVA, *p* < 0.01; ***—significance of ANOVA, *p* < 0.001; T2D—performance score (discharge + change value); side differences—significance *p* and effect size η_p_^2^ of differences in side for bilaterally GAMS items; group difference—significance *p* and effect size η_p_^2^ for differences in between groups in GAMS items.

## References

[1] Ramesh S.H., Lemaire E.D., Tu A., Cheung K., Baddour N. (2023). Automated Implementation of the Edinburgh Visual Gait Score (EVGS) Using OpenPose and Handheld Smartphone Video. Sensors.

[2] Pinto R.F., Birmingham T.B., Leitch K.M., Atkinson H.F., Jones I.C., Giffin J.R. (2020). Reliability and validity of knee angles and moments in patients with osteoarthritis using a treadmill-based gait analysis system. Gait Posture.

[3] Wren T.A.L., Tucker C.A., Rethlefsen S.A., Gorton G.E., Ounpuu S. (2020). Clinical efficacy of instrumented gait analysis: Systematic review 2020 update. Gait Posture.

[4] Naili J.E., Esbjornsson A.C., Iversen M.D., Schwartz M.H., Hedstrom M., Hager C.K., Brostrom E.W. (2017). The impact of symptomatic knee osteoarthritis on overall gait pattern deviations and its association with performance-based measures and patient-reported outcomes. Knee.

[5] Langley B., Greig M. (2023). The gait abnormality index: A summary metric for three-dimensional gait analysis. Gait Posture.

[6] Dürregger C., Adamer K.A., Pirchl M., Fischer M.J. (2020). Inter-rater reliability of a newly developed gait analysis and motion score. J. Orthop. Trauma Rehabil..

[7] Pension Insurance Institution [Pensionsversicherungsanstalt] (2025). Medical Performance Profile for Inpatient Rehabilitation (MLP STAT).

[8] Faude O., Donath L., Roth R., Fricker L., Zahner L. (2012). Reliability of gait parameters during treadmill walking in community-dwelling healthy seniors. Gait Posture.

[9] Walker L.C., Clement N.D., Bardgett M., Weir D., Holland J., Gerrand C., Deehan D.J. (2018). The WOMAC score can be reliably used to classify patient satisfaction after total knee arthroplasty. Knee Surg. Sports Traumatol. Arthrosc..

[10] Podsiadlo D., Richardson S. (1991). The timed “Up & Go”: A test of basic functional mobility for frail elderly persons. J. Am. Geriatr. Soc..

[11] Steffen T.M., Hacker T.A., Mollinger L. (2002). Age- and gender-related test performance in community-dwelling elderly people: Six-Minute Walk Test, Berg Balance Scale, Timed Up & Go Test, and gait speeds. Phys. Ther..

[12] Sawilowsky S.S. (2009). New Effect Size Rules of Thumb. J. Mod. Appl. Stat. Methods.

[13] Cohen J. (1988). Statistical Power Analysis for the Behavioral Sciences.

[14] Hinkle D.E., Wiersma W., Jurs S.G. (2003). Applied Statistics for the Behavioral Sciences.

[15] Bily W., Jauker J., Nics H., Grote V., Pirchl M., Fischer M.J. (2022). Associations between Patient-Reported and Clinician-Reported Outcome Measures in Patients after Traumatic Injuries of the Lower Limb. Int. J. Environ. Res. Public Health.

[16] Wagner B., Zdravkovic A., Pirchl M., Puhan M.A., Zwick R.H., Grote V., Crevenna R., Fischer M.J. (2022). Performance Score (T2D)-A New Perspective in the Assessment of Six-Minute Walking Tests in Pulmonary Rehabilitation. Diagnostics.

[17] Zdravkovic A., Grote V., Pirchl M., Stockinger M., Crevenna R., Fischer M.J. (2022). Comparison of patient- and clinician-reported outcome measures in lower back rehabilitation: Introducing a new integrated performance measure (t2D). Qual Life Res..

[18] Tu Y.K., Gilthorpe M.S. (2007). Revisiting the relation between change and initial value: A review and evaluation. Stat. Med..

[19] Wright A.A., Cook C.E., Baxter G.D., Dockerty J.D., Abbott J.H. (2011). A comparison of 3 methodological approaches to defining major clinically important improvement of 4 performance measures in patients with hip osteoarthritis. J. Orthop. Sports Phys. Ther..

[20] Angst F., Aeschlimann A., Michel B.A., Stucki G. (2002). Minimal clinically important rehabilitation effects in patients with osteoarthritis of the lower extremities. J. Rheumatol..

[21] Christopher A., Kraft E., Olenick H., Kiesling R., Doty A. (2021). The reliability and validity of the Timed Up and Go as a clinical tool in individuals with and without disabilities across a lifespan: A systematic review. Disabil. Rehabil..

[22] Zhang Y., Ren J., Zang Y., Guo W., Disantis A., Martin R.L. (2023). Cross-Culturally Adapted Versions of Patient Reported Outcome Measures for the Lower Extremity. Int. J. Sports Phys. Ther..

[23] Maity S., Das H., Chakrawarty A., Devanbu V.G.C. (2024). Gait analysis and geriatric syndromes: An association among elderly patients attending a teaching hospital of Delhi. J. Family Med. Prim. Care.

[24] Zhao R., Wei X., Hu S., Zhang Y., Wu H., Li P., Zhao Y. (2024). Deficient gait function despite effect index of the Western Ontario and McMaster university osteoarthritis index score considered cured one year after bilateral total knee arthroplasty. BMC Musculoskelet Disord.

[25] Liebensteiner M.C., Herten A., Gstoettner M., Thaler M., Krismer M., Bach C.M. (2008). Correlation between objective gait parameters and subjective score measurements before and after total knee arthroplasty. Knee.

[26] Nebel M.B., Sims E.L., Keefe F.J., Kraus V.B., Guilak F., Caldwell D.S., Pells J.J., Queen R., Schmitt D. (2009). The relationship of self-reported pain and functional impairment to gait mechanics in overweight and obese persons with knee osteoarthritis. Arch. Phys. Med. Rehabil..

[27] Bolink S.A., Grimm B., Heyligers I.C. (2015). Patient-reported outcome measures versus inertial performance-based outcome measures: A prospective study in patients undergoing primary total knee arthroplasty. Knee.

[28] Li H., Hu S., Zhao R., Zhang Y., Huang L., Shi J., Li P., Wei X. (2022). Gait Analysis of Bilateral Knee Osteoarthritis and Its Correlation with Western Ontario and McMaster University Osteoarthritis Index Assessment. Medicina.

[29] Kirschberg J., Goralski S., Layher F., Sander K., Matziolis G. (2018). Normalized gait analysis parameters are closely related to patient-reported outcome measures after total knee arthroplasty. Arch. Orthop. Trauma Surg..

[30] Bonnefoy-Mazure A., Armand S., Sagawa Y., Suvà D., Miozzari H., Turcot K. (2017). Knee Kinematic and Clinical Outcomes Evolution Before, 3 Months, and 1 Year After Total Knee Arthroplasty. J. Arthroplast..

[31] Senden R., Grimm B., Meijer K., Savelberg H., Heyligers I.C. (2011). The importance to including objective functional outcomes in the clinical follow up of total knee arthroplasty patients. Knee.

[32] Turcot K., Sagawa Y., Fritschy D., Hoffmeyer P., Suvà D., Armand S. (2013). How gait and clinical outcomes contribute to patients’ satisfaction three months following a total knee arthroplasty. J. Arthroplast..

